# Supplementary material for: Microbiome Analysis Reveals Biocontrol of Aspergillus and Mycotoxin Mitigation in Maize by the Growth-Promoting Fungal Endophyte Colletotrichum tofieldiae Ct0861
Source: Plants (Basel). 2025 Oct 22;14(21):3236. doi: 10.3390/plants14213236 (PMC12608721; doi:10.3390/plants14213236)
Supplement: Supplementary file 1 [file plants-14-03236-s001.zip › Additional File 1_Supplementary methods_r1.pdf]

Microbiome analysis reveals biocontrol of *Aspergillus* and mycotoxin mitigation in maize by the growth-promoting fungal endophyte *Colletotrichum tofieldiae* Ct0861

Sandra Díaz-González, Carlos González-Sanz, Sara González-Bodí, Patricia Marín, Frédéric Brunner and Soledad Sacristán

Additional File 1: Supplementary Methods

1. **Irrigation program for open-field maize plants.**

	CROP ESTABLISHMENT	VEGETATIVE DEVELOPMENT		REPRODUCTIVE STAGE	SENESCENCE		
		Stage 1	Stage 2		Stage 1	Stage 2	Stage 3
No. days per period	20	17	18	40	10	10	10
Watering regime until	28/05/18	14/06/18	02/07/18	11/08/18	21/08/18	31/08/18	10/09/18
Kc[1]	0.4	0.79	1.20	1.20	0.92	0.63	0.35
Eto (mm/day)	4.80	5.25	5.72	5.55	5.16	4.73	4.32
Rainfall (mm/day)	0.30	0.14	0.20	0.10	0.29	0.35	0.62
Effective rainfall (mm/day)	0.1	0.0	0.0	0.0	0.1	0.1	0.3
ETC (mm/day)	1.92	4.14	6.86	6.66	4.73	2.99	1.51
GIWR (mm/day)	1.82	4.14	6.84	6.66	4.64	2.86	1.17
NIWR (mm/day)	1.80	4.10	6.77	6.60	4.59	2.83	1.16
TIWR (mm/day)	2.37	5.39	8.91	8.68	6.04	3.72	1.53
Watering time (h/day)	0.51	1.16	1.91	1.86	1.29	0.80	0.33
Watering time in OW (min/day)	30	69	115	112	78	48	20

Eto	Reference evapotranspiration
ETC	Reference crop evapotranspiration
GIWR	Gross irrigation water requirements
NIWR	Net irrigation water requirements
TIWR	Total irrigation water requirements

Calculations were made on long-term data from the closest climate station, located in San Javier (37° 47' 20" N, 0° 48' 12" O; IMIDA 2018).

[1]Corn Kc was adapted from CROPWAT (version 8.0) and seed breeder information.

Reference

IMIDA (Instituto Murciano de Investigación y Desarrollo Agrario y Alimentario). Data from: Sistema de Información Agrometeorológica de la Región de Murcia (SIAM). Available at: http://siam.imida.es/apex/f?p=101:46:1821222586473642. (Accessed April 1, 2018).

2. Processing of soil and plant material and DNA extraction

<u>BULK SOIL COMPARTMENT</u>: Approximately 0.5 L of soil material from the top 30 cm surrounding layer of each plant were collected in a plastic zip bag. Then, 100 mg were directly transferred to a 2 mL Eppendorf tube with lysis matrix from FastDNA™ Spin Kit for Soil (MP Biomedicals).

<u>RHIZOSPHERE COMPARTMENT</u>: Roots were separated from the rest of the plant and shaken to remove the excess of soil. Roots were washed twice in two 500 mL plastic recipients with 100 mL of sterile distilled water to remove attached soil particles. The 200 mL of washing water (containing soil particles) from the same plant, were mixed and 30 mL were transferred to a 50 mL falcon. Falcon tubes were centrifuged at 4,000xg for 15 min. After centrifugation, 95% of supernatant was discarded. Pellet was resuspended into the remaining supernatant. Using a cut P1000 tip, 300 µL of the mixture were transferred to a 2 mL tube with lysis matrix from FastDNA™ Spin Kit for Soil (MP Biomedicals).

<u>ROOT COMPARTMENT</u>: Roots were gently washed with tap water in order to remove any remaining soil residue. Roots were cut into small sections with pruning shears, which were disinfected with a 20% bleach solution between samples. Root sections were wrapped in aluminum foil for their storage.

<u>LEAF COMPARTMENT</u>: Four circles of ~1 cm of diameter were collected from the youngest unfolded leaf of each plant and introduced in a 2 mL Eppendorf tube filled with sterile glass beads (2.7 mm Ø, Carl Roth GmbH & Co.).

<u>GRAIN COMPARTMENT</u>: Grain samples were only collected for 4MPS. Mature cobs from the same plant were manually shelled, grains were pooled and transferred to 15 mL falcon.

All samples were kept at -80ºC until DNA extraction.

Bulk soil, rhizosphere, and leaf material were homogenized using FastPrep-24™ 5G homogenizer (MP Biomedicals), while roots and grain samples were disrupted with mortar and liquid nitrogen.

DNA was extracted from bulk soil, rhizosphere, and grain samples with FastDNA™ Spin Kit for Soil (MP Biomedicals) following the manufacturer's instructions. DNA extraction from root and leaf tissues was conducted with DNeasy Plant Mini Kit (Qiagen), following the manufacturer's instructions with a minor adaptation in elution volumes (30 µL instead of 100 µL). Centrifugation steps were carried out at 4ºC. DNA quality was verified by agarose electrophoresis (1.5% agarose, Agarose D1 Medium EEO, Conda Pronadisa). DNA quantification was carried out with Quant-iT™ PicroGreen™ dsDNA Assay Kit (Invitrogen) in Varioskan™ LUX Multimode microplate reader (Thermo Scientific).
